# Ferritin nanovehicle for targeted delivery of cytochrome C to cancer cells

**DOI:** 10.1038/s41598-019-48037-z

**Published:** 2019-08-13

**Authors:** Alberto Macone, Silvia Masciarelli, Federica Palombarini, Deborah Quaglio, Alberto Boffi, Matilde Cardoso Trabuco, Paola Baiocco, Francesco Fazi, Alessandra Bonamore

**Affiliations:** 1 Department of Biochemical Sciences “Alessandro Rossi Fanelli”, Sapienza University of Rome, Pizzale Aldo Moro 5, 00185 Rome, Italy; 2 Department of Anatomical, Histological, Forensic & Orthopaedic Sciences, Section of Histology and Medical Embryology, Sapienza University of Rome, laboratory affiliated to Istituto Pasteur Italia-Fondazione Cenci Bolognetti, Via A. Scarpa, 14-16, 00161 Rome, Italy; 3 Department of Chemistry and Technology of Drugs, Sapienza University of Rome, Pizzale Aldo Moro 5, 00185 Rome, Italy; 4 Center for Life Nano Science @ Sapienza, Italian Institute of Technology, Viale Regina Elena 291, Rome, 00161 Italy

**Keywords:** Carrier proteins, Cell delivery, Nanoparticles

## Abstract

In this work, we have exploited the unique properties of a chimeric archaeal-human ferritin to encapsulate, deliver and release cytochrome c and induce apoptosis in a myeloid leukemia cell line. The chimeric protein combines the versatility in 24-meric assembly and cargo incorporation capability of *Archaeglobus fulgidus* ferritin with specific binding of human H ferritin to CD71, the “heavy duty” carrier responsible for transferrin-iron uptake. Delivery of ferritin-encapsulated cytochrome C to the Acute Promyelocytic Leukemia (APL) NB4 cell line, highly resistant to transfection by conventional methods, was successfully achieved *in vitro*. The effective liberation of cytochrome C within the cytosolic environment, demonstrated by double fluorescent labelling, induced apoptosis in the cancer cells.

## Introduction

Therapeutic proteins are emerging as a new important class of drugs employed to treat some high-incidence human diseases including cancer, metabolic disorders, autoimmune diseases, etc. Many proteins from diverse natural sources have been tested for their cytotoxic activity toward mammalian cells. Among them plant, microbial and animal toxins have been widely tested^1^ for their potential cytotoxic activity. In this frame, proapoptotic proteins are gaining importance as therapeutic candidates in cancer therapy. However, the effects of these proteins are often limited by their inability to penetrate mammalian cell membranes. To date, various nanovehicles have been explored to facilitate intracellular delivery of bioactive proteins for therapeutic purposes, including liposomes, bio-organic polymers, gold and silica nanoparticles, quantum dots and carbon nanotubes^2,3^. However, no single formulation has been proven to be ideal for all types of bioactive proteins as extensive manipulations are necessary to confer both protein loading capabilities and selective cell targeting properties. Moreover, once the protein carrier has been uptaken by the targeted cell, it must escape during early stages of the endo-lysosomal pathway to avoid degradation due to low pH values and hydrolytic enzymes action in the lysosomes^2^. Thus far, novel strategies are being explored to expand the scope of protein delivery and move forward the application of innovative protein therapeutics to clinical settings.

Ferritin proteins have been taking center stage in recent years as smart nanocarriers for drug delivery due to their hollow cage-like structures and their unique 24-meric assembly^4–9^. Human H ferritins are naturally targeted toward TfR1 (or CD71) receptor highly expressed in iron avid, fast replicating, tumor cells^10^. Thus, ferritin based protein cages have been developed as versatile platforms for multiple applications in nanomedicine. Virtually all proposed applications rely in the delivery of small therapeutic molecules or metal labels encapsulated within the human H-ferritin homopolymer within a procedure that entails subunit dissociation of the ferritin 24-mer at extreme pH values (<2.0 or >10.0) followed by neutralization and subsequent encapsulation of the small molecule of interest. Such procedure, however, is not amenable to the encapsulation of most proteins that display both acid or alkaline folding instability. In contrast, the ferritin from *Archaeglobus fulgidus* (AfFt) has emerged as an alternative to human ferritin homopolymers as it requires mild cargo material encapsulation conditions in view of the unique self-assembly properties that entail divalent cation driven assembly at neutral pH values^11^. AfFt assembles in a distinctive tetrahedral geometry as a result of a particular packing between four hexameric units into a unique 24-mer structure, which results in the formation of four wide triangular pores (45 Å) on the protein shell^12,13^. As such, AfFt has been proven to be the ideal scaffold to host small, basic proteins within the internal cavity in a reversible manner^14^. Notable examples have been reported in very recent literature in which native *Archaeglobus* ferritin has been shown to be able to reversibly encapsulate and maintain catalytically-active enzymes or proteins like GFP^15^. *Archaeglobus fulgidus* ferritin was further engineered by grafting a 12 aminoacid loop (BC loop in ferritin topology), typical of H-ferritin homopolymer, into the archaeal ferritin itself. The resultant chimeric protein, referred to as “humanized Archaeoglobus ferritin” (HumFt), was shown to be capable of specific interaction with the extracellular moiety epitopes of the CD71 receptor of target cells in a similar way as the human H ferritin^16^. The engineered HumFt thus combines the versatility in assembly and cargo incorporation properties of AfFt with binding capabilities and cellular uptake properties of human H homopolymer. As such, HumFt represents a uniquely suitable scaffold for incorporating a wealth of diverse substructures inside the protein cavity, either by assembly/disassembly process at neutral pH or by diffusion through the large triangular pores on the surface. The reversible assembly/disassembly dynamics also indicate the possibility of release of the payload within the cytosol.

In the present paper, we exploited the unique properties of HumFt to encapsulate and deliver bioactive full-length cytochrome C to tumor cells in order to trigger an apoptotic response.

Cytochrome C is a small protein (104 aminoacids) with distinct basic properties (pI = 10.8) due to the presence of excess positive charges necessary for the interaction with the terminal oxidase in mitochondria as well as with the apoptotic protease activating factor-1 (Apaf-1) in the cytosol^17,18^. This last interaction triggers apoptosome assembly and caspase cascade that eventually lead to cell death^19^.

## Results

**Figure 1:**
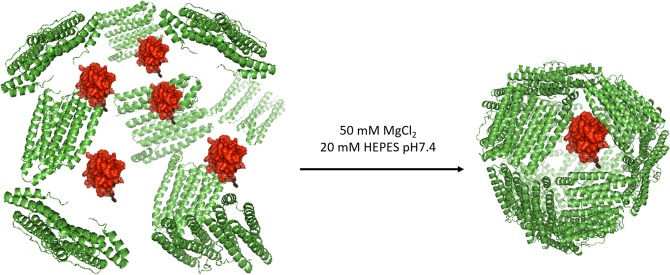
HumFt-Cyt C complex formation. HumFt was dissociated at low ionic strength and re-associated by adding MgCl_2_ in the presence of cytochrome C. In these experimental conditions Cyt C is encapsulated inside the protein cavity.

Cytochrome C is roughly a parallelepiped (2.1 × 2.5 × 3.7 nm) with a volume of about 20 nm^3^, a dimension that allows it to fit even in multiple copies within the negatively charged, 350 nm^3^ wide HumFt internal cavity (Fig. 1). Encapsulation of positively charged cytochrome C into HumFt has been achieved by mixing the two proteins at neutral pH in the absence of divalent cations and subsequent addition of MgCl_2_ in order to promote ferritin assembly. In this condition HumFt was able to stably encapsulate cytochrome C although with ratios lower than the expected (Cyt C:HumFt = 0.43 ± 0.14). A specific chemical modification of the HumFt internal cavity Cys54 residue with iodoactic acid was then introduced in order to increase the negative charge of the internal surface and favor electrostatic interactions with the positively charged cytochrome C. To allow the correct assembly of the cage, S-carboxymethylation reaction has to be strictly controlled. In fact, the protein was correctly assembled as a 24-mer only when S-carboxymethylation occured at no more than 50% of the available cysteine residues (12 extra negative charges added). This result was obtained testing different concentration of iodoacetic acid and evaluating the protein assembly by High Performance Size Exclusion Chromatography (HP-SEC) (see Figs S1 and S2).

**Figure 2:**
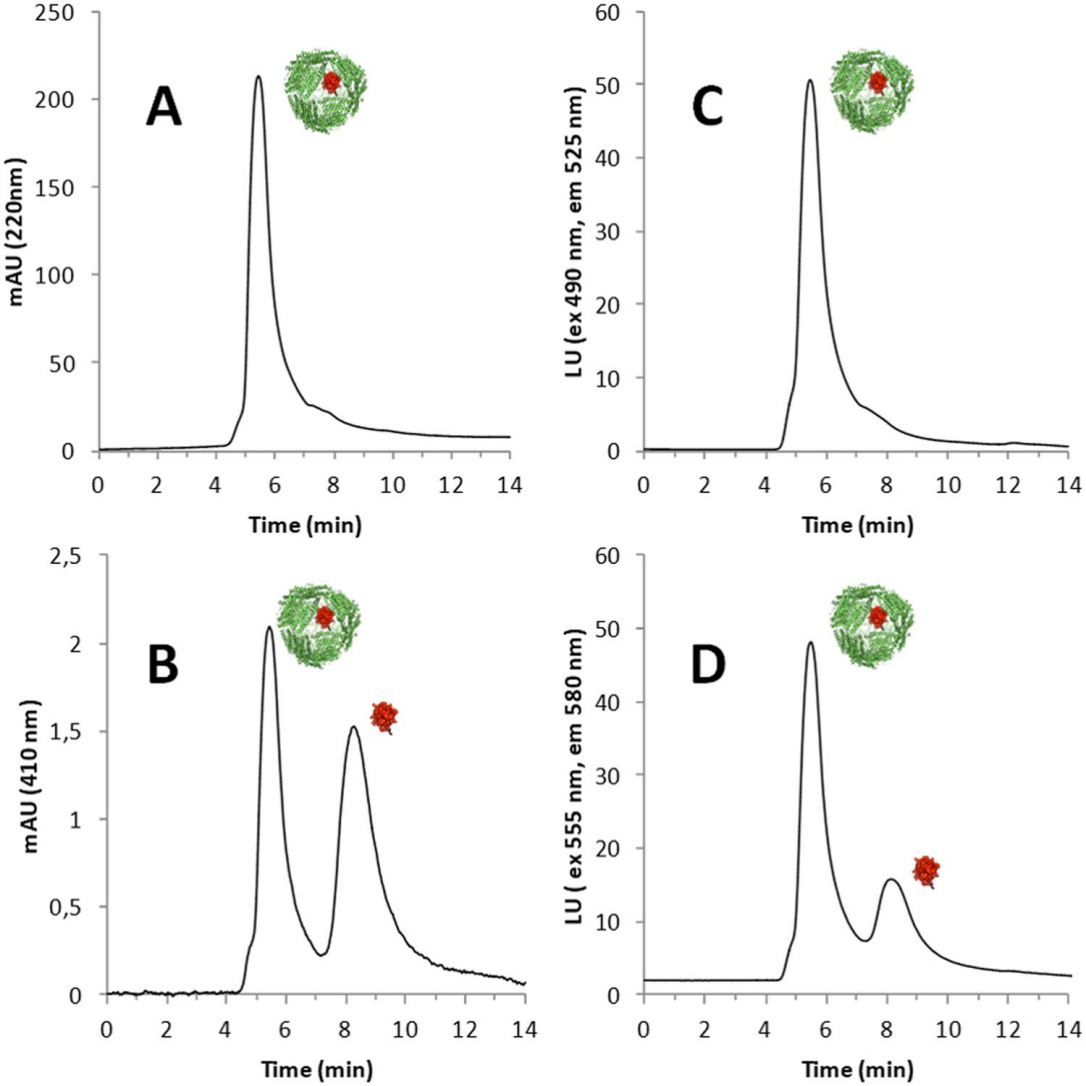
HP-SEC of the complex S-CMHumFt-FITC and Cyt C-Alexa Fluor 555. (**A**) Elution profile of the complex followed at 220 nm: the main peak (5.8 min) corresponds to ferritin 24-mer. (**B)** Elution profile of the complex followed at 410 nm. At this wavelength, corresponding to the maximum absorption of Cyt C, two peaks can be detected: the first one (5.8 min) is due to Cyt c encapsulated inside the ferritin cavity (empty S-CMHumFt shows no signal at this wavelength) and the second one (8.3 min) corresponds to free Cyt C. Free Cyt C concentration is always lower than the encapsulated one. (**C)** Elution profile of the complex with ferritin labelled with fluoresceine (λ_ex_ = 490 nm, λ_em_ = 525 nm). (**D)** Elution profile of the complex with Cyt C labelled with Alexa Fluor 555 (λ_ex_ = 555 nm, λ_em_ = 580 nm). Even in this case two peaks can be detected: the first one (5.8 min) is due to Alexa Fluor 555 labeled Cyt C encapsulated inside the ferritin cavity (empty S-CMHumFt shows no signal at this wavelength) and the second one (8.3 min) corresponds to free labeled Cyt C.

HP-SEC analysis with UV-vis and fluorimetric detection was used to study the ferritin-cytochrome C complex formation, by labelling these proteins with two different fluorescent probes: cytochrome C was labeled with AlexaFluor 555 NHS (Cyt C 555) and carboxymethylated humanized ferritin (S-CMHumFt) with fluorescein isothiocyanide (S-CMHumFt-FITC). As shown in Fig. 2, Cyt C is stably incorporated within the internal cavity of S-CMHumFt. In fact, the typical Soret band of the cyt C porphyrin group (410 nm) can be detected at the retention time of ferritin 24-mer (5.3 min), while free cytochrome C elutes at 8.3 min (Fig. 2, panel C). The encapsulation was further confirmed following the fluorescence emission of AlexaFluor labelled cytochrome C (Fig. 2, panel D). The quantitative analysis of the chromatograms proves that carboxymethylation of cysteine residues favored the interaction between the proteins leading to a 3.2 fold increase in cytochrome C encapsulation with respect to the unmodified protein (Cyt C:HumFt = 1.4 ± 0.27). In summary, the set of HP-SEC measurements clearly indicated that after mixing S-CMHumFt with a 20 fold molar excess of Cyt C and subsequent addition of MgCl_2_ (50 mM), a fully assembled ferritin 24-mer containing Cyt C inside the cavity is obtained. Under these conditions, the preparation was stable over 1 month, sterile filtered at 4 °C (Fig. S3).

**Figure 3:**
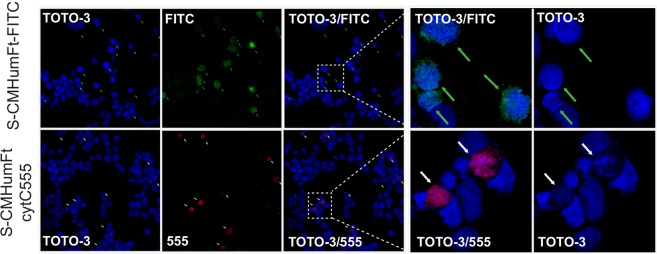
Confocal microscopy of APL NB4 cells treated with S-CMHumFt-FITC and S-CMHumFt-Cyt C 555. Cytochrome C labeled with AlexaFluor 555 is delivered into the APL NB4 cell line by S-CMHumFt leading to apoptotic cell death. NB4 cells were cultured in the presence of 150 μg/mL S-CMHumFt-FITC (upper panels) or of 150 μg/mL S-CMHumFt loaded with cytochrome C-AlexaFluor 555 (lower panels) for 24 hours and analyzed by confocal microscopy, after labeling the DNA with the TOTO-3 dye. All the cells that internalized detectable amounts of Cyt C 555 presented apoptotic nuclei as indicated by nuclear condensation (white arrows). On the contrary, even the cells that internalized high amounts of S-CMHumFt-FITC alone showed vital, uncondensed nuclei (green arrows). The panels on the right show an enlargement of the cells into the insets.

In order to assess the ability of S-CMHumFt to deliver cytochrome C to human cells and consequently induce apoptosis, the Acute Promyelocytic Leukemia (APL) cell line NB4^20^, a subtype of Acute Myeloid Leukemia (AML) was used. NB4 myeloid cells have been chosen mainly for two reasons: i) they are particularly difficult to transfect with conventional liposome based methods and, to date, the only successful delivery methods rely on lentiviral particles infection or nucleofection; ii) AML primary blasts do express high levels of CD71^21,22^. In the first step, the ability of NB4 cells to incorporate humanized ferritin was investigated. Figure 3 (upper panel) shows the uptake of S-CMHumFt labeled with Fluorescein isothiocyanate (S-CMHumFt-FITC) by NB4 cells, thus confirming previous investigations on the internalization and cytosolic and perinuclear distribution of the same protein in HeLa cells^16^. When NB4 cells were treated with S-CMHumFt containing Cyt C 555, a clear fluorescent signal was observed in the cytosolic environment within 24 hours from incubation of the complex (Fig. 3, lower panel). Furthermore, after 24 hours, a significant fraction of the cells that incorporated Cyt C 555 underwent apoptosis as indicated by chromatin condensation and by uptake of the vital dye Sytox Blue (Figs 3 and S4). After 72 hours most of the NB4 cells incubated with S-CMHumFt-Cyt C 555 underwent cell death (Fig. S4). Control experiments carried out within the same time frames by using empty unlabeled and FITC labelled S-CMHumFt did not show significant alterations. The same was observed treating NB4 cells with the same amount of free unlabeled and AlexaFluor 555 labelled Cyt C.

## Discussion

In the present study, we report cytochrome C delivery to Acute Promyelocytic Leukemia (APL) NB4 cell line by means of a chimeric archeal-human ferritin (HumFt) that combines the unique salt mediated association/dissociation equilibrium of *Archaeoglobus fulgidus* ferritin with the human H ferritin ability of recognizing CD71 receptor. Human ferritin has been widely used to entrap a number of compounds. Nevertheless, given the high stability of the quaternary structure, the dissociation process requires harsh conditions, such as acidic or basic pH values. Under these conditions, only pH resistant compounds can be loaded and the protein recovery is often very low. The use of HumFt, instead, allows disassembly into subunits by just changing the ionic strength. In this way, a plethora of payloads including therapeutic proteins may be easily encapsulated with high efficiency. The chimeric HumFt, moreover, is able to target CD71. This receptor is overexpressed in many tumors including AML cells, that are highly resistant to transfection with conventional methods.

In order to insert extra negative charges inside the cavity to favor the encapsulation of positively charged proteins, the chimeric ferritin carrier has been further modified by means of a carboxyemthylation reaction, leading to the formation of S-CMHumFt. The results here presented show that such chemical modification is indeed able to increase the number of cyt C molecules incapsulated per cage. Cytochrome C delivery to target cells has been demonstrated by Alexa Fluor 555 labelling. Our results show that S-CMHumFt acts as a “Trojan horse” by releasing cytochrome C into AML cells and inducing apoptosis. The complex between S-CMHumFt and cytochrome C appears as a unique system as it is capable of efficient CD71 receptor targeting. Previous investigations in cytochrome C delivery were in fact based on efficient, but untargeted, delivery systems^23–27^.

Ferritins have recently been tested as nanocages for enzyme encapsulation^14^ but, to the best of our knowledge, this is the very first report of a ferritin-mediated cell targeted delivery of a therapeutic protein. This is even more striking if one considers the possibility of introducing enhanced pro-apoptotic variants of human cytochrome C^28^, currently under scrutiny.

Humanized ferritin represents a powerful model system in that it could be further tailored according to the expanding catalogues of cytotoxic proteins and peptides (venoms, toxins, proaoptotic proteins, etc.) that are emerging as a new generation of antitumor drugs. Ferritin, in fact, can be considered a versatile tool that could be chemically functionalized and/or genetically engineered to expand the selectivity toward different protein cargos.

## Materials and Methods

All salts and buffers, iodoacetic acid, fluorescein isothiocyanate, hydroxymercuribenzoate and cytochrome C from equine heart were purchased from Sigma Aldrich. Alexa Fluor 555 was purchased from ThermoFisher Scientific.

### S-Carboxymethylation of HumFt and –SH titration

*A. fulgidus* humanized ferritin (HumFt) was expressed in *E. coli*, purified and quantified as previously described^16^. S-Carboxymethylation of cysteine residues has been performed using iodoacetic acid as alkylating agent^29^. 10 µL 1 M dithiothreitol were added to 1 mL of HumFt 1 mg/mL in 0.1 M HEPES buffer pH 8.4 and the reduction reaction was kept for 2 hours at room temperature. 12 µL of 1 M iodoacetic acid in 1 M NaOH were added to the mixture and kept in the dark at room temperature for 30 minutes. The reaction was quenched adding 20 µL 1 M dithiothreitol. The excess of reagents was removed using 30 kDa Amicon Ultra-15 centrifugal filters (Merck Millipore) exchanging the buffer with 20 mM HEPES buffer pH 7.4 containing 50 mM MgCl_2_. The modified protein was sterile filtered and stored at 4 °C.

Thiol groups titration was performed as described by Boyer *et al*.^30^. Briefly, 4.2 mg of p-hydroxymercuribenzoate (pOHMB) were dissolved in 1 mL of NaOH 0.5 M. The resulting solution was diluted 1:10 with phosphate buffer 0.1 M pH 7.0. The stock concentration was determined by UV detection at 232 nm using a molar extinction coefficient of 16900 M^−1^ cm^−1^ and adjusting the concentration at 1 mM. S-CMHumFt, treated with iodoacetic acid as previously described, was diluted in phosphate buffer 0.1 M pH 7.0 to a final concentration of 20 μM in monomer. The number of unreacted free thiol groups was determined by adding 2 μL aliquots of 1 mM pOHMB to a solution of 20 µM S-CMHumFt and measuring the mercaptide formation following the absorbance increase at 250 nm.

### Protein labeling

S-CMHumFt was labeled with Fluorescein-isothiocyanide (FITC) (Sigma Aldrich) according to the manufacturer’s standard protocol. Briefly, 2 mg/mL of purified protein were equilibrated in carbonate buffer pH 9.0. 50 µL of FITC 1 mg/mL in DMSO were added to 1 mL of S-CMHumFt solution and the reaction mix was stirred for 2 hours at room temperature. The non-reacted dye was removed by gel filtration chromatography and the labeled protein was dialyzed versus 20 mM HEPES buffer pH 7.4 containing 50 mM MgCl_2_.

Equine hearth cytochrome C (Cyt C) (Sigma Aldrich) was labeled with Alexa Fluor 555 NHS Ester (Succinimidyl Ester) (Thermo Fisher Scientific) according to the manufacturer’s standard protocol. Briefly, 10 mg of the protein were dissolved in 1 mL 0.1 M sodium bicarbonate buffer pH 8.4. 1 mg of Alexa Fluor 555 NHS Ester dissolved in 0.1 mL of DMSO was added dropwise to cytochrome C solution. The reaction was incubated in the dark for 1 hour at room temperature with continuous stirring. The non-reacted dye was removed by gel filtration chromatography and the labeled protein was dialyzed versus 20 mM HEPES buffer pH 7.4 containing 100 mM MgCl_2_.

Protein labeling was checked by High Performance Size Exclusion Chromatography (HP-SEC).

### S-CMHumFt-Cyt C complex preparation

S-CMHumFt was dialyzed versus 20 mM HEPES buffer pH 7.4 without MgCl_2_ to allow the 24-mer to dimer transition. Afterwards, 2 mL S-CMHumFt 1.5 mg/mL (3 µM 24-mer) were added dropwise to 2 mL Cyt C 0.8 mg/mL (64 µM) in 20 mM HEPES buffer pH 7.4 containing 100 mM MgCl_2_. After mixing, MgCl_2_ concentration (50 mM) shifts the equilibrium towards the ferritin associated form (24-mer) containing Cyt C inside the cavity.

Complex formation with labeled proteins was carried out in the same condition described above.

S-CMHumFt-Cyt C complex formation (labeled and unlabeled) was monitored by HP-SEC.

The stability of the complex was checked over time (1 month) by HP-SEC, keeping the sample sterile filtered at 4 °C.

### High performance size exclusion chromatography

HP-SEC was performed using an Agilent Infinity 1260 HPLC apparatus equipped with UV and fluorometric detectors. Separation was carried out using an Agilent AdvanceBio SEC. 300 Å, 7.8 × 150 mm, 2.7 µm, LC column connected to an AdvanceBio SEC. 300 Å, 7.8 × 50 mm, 2.7 µm, LC guard column. Isocratic analysis was carried out with 20 mM HEPES buffer pH 7.4, 50 mM MgCl_2_ as mobile phase. Flow rate was 0.7 mL/min over an elution window of 14 min.

S-CMHumFt elution was followed using the UV detection at 220 nm. In its fluorescein labeled derivative the protein was also detected with fluorimetric detector set to excitation and emission wavelengths of 490 and 525 nm, respectively.

Cyt C elution was followed using the UV detection at 410 nm. In its Alexa Fluor labeled derivative the protein was also detected with fluorimetric detector set to excitation and emission wavelengths of 555 and 580 nm, respectively.

S-CMHumFt-Cyt C complex formation was checked following the typical cytochrome C signals at the retention time of ferritin 24-mer.

Protein standards (highly purified ferritin and cytochrome c) were prepared in the same solution as the mobile phase. Ferritin concentration was determined using the theoretical ε_280_ = 32430 M^−1^ cm^−1^. The calibration curves for ferritin and cytochrome C were constructed with humanized ferritin standard concentrations in the range 0.5 to10 mg/mL and cytochrome C standard concentrations in the range 0.05 to 5 mg/mL.

### *In vitro* cell studies

The human acute promyelocytic leukemia cell line NB4 was obtained by DSMZ (Branuschweig, Germany). The cells were cultured and treated in RPMI 1640 medium with the addition of penicillin/streptomycin and 10% FCS (Gibco, ThermoFisher Scientific, Waltham, MA, USA).

NB4 cells were treated with 150 μg/mL S-CMHumFt, S-CMHumFt-FITC or S-CMHumFt loaded with cytochrome C-AlexaFluor 555 (S-CMHumFt-Cyt C 555) prepared as described above for the time points indicated in the figures. Since some free cytochrome C-AlexaFluor 555 was measured in the preparation of S-CMHumFt-Cyt C 555, a sample of cells was treated with the same concentration of free cytochrome C-AlexaFluor 555 (indicated in Fig. S4 as CytC 555).

Cell death and uptake of S-CMHumFt-FITC or of CytC 555 was assessed by flow cytometry (CyAN ADP DAKO). At the indicated times of incubation with the different compounds, 2 × 10^5^ cells for each experimental point were collected, washed in PBS (Gibco, ThermoFisher Scientific) and resuspended in PBS in the presence of 1 μM Sytox Blue Dead Cell Stain (Molecular Probes, ThermoFisher Scientific, Waltham, MA, USA) for 15 minutes before flow cytometry analysis.

For confocal immunofluorescence analysis 3 × 10^5^ cells for each point, treated as described above for 24 hours were laid on glass slides by cytospin centrifugation (Shandon, ThermoFisher Scientific, Waltham, MA, USA) and fixed in 4% PFA for 10 minutes; DNA was counterstained with TOTO-3 (Molecular Probes, ThermoFisher Scientific, Waltham, MA, USA) and slides mounted with Vectashield mounting medium (Vector Laboratories, Burlingame, CA, USA). Confocal images were acquired with a Leica laser scanning microscope TCS SP2 equipped with Leica Confocal Software (Leica, Milano, Italy).

## Supplementary information

Ferritin nanovehicle for targeted delivery of cytochrome C to cancer cells
