# Supplementary material for: Ferritin nanovehicle for targeted delivery of cytochrome C to cancer cells
Source: Sci Rep. 2019 Aug 13;9:11749. doi: 10.1038/s41598-019-48037-z (PMC6692331; doi:10.1038/s41598-019-48037-z)
Supplement: Supplementary file 1 — Ferritin nanovehicle for targeted delivery of cytochrome C to cancer cells [file 41598_2019_48037_MOESM1_ESM.docx]

**SUPPORTING INFORMATION**

**Ferritin nanovehicle for targeted delivery of cytochrome C to cancer cells**

Alberto Macone, Silvia Masciarelli, Federica Palombarini, Deborah Quaglio, Alberto Boffi, Matilde Cardoso Trabuco, Paola Baiocco, Francesco Fazi, and Alessandra Bonamore

**S-Carboxymethylation of HumFt and –SH titration**


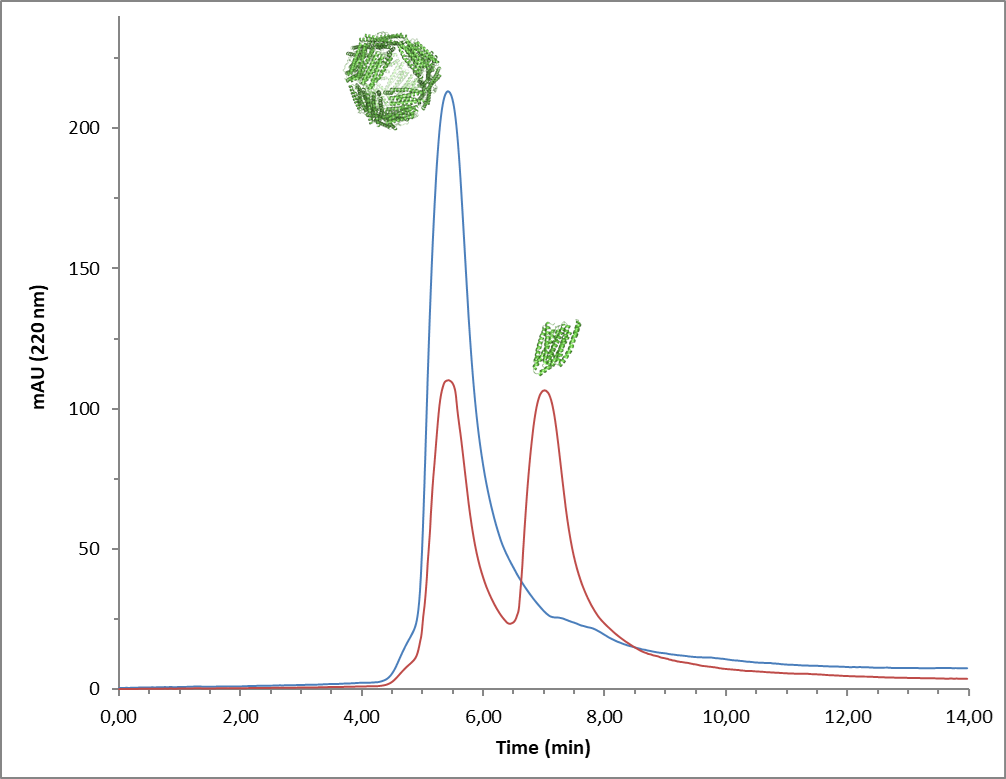


Figure S1: HP-SEC analysis of S-CMHumFt treated with different amount of iodoacetic acid. In red the elution profile of ferritin treated with large excess of iodacetic acid (25 mM). In blue the elution profile of ferritin treated with iodoacetic acid in the concentration described above (12 mM). In this last condition S-CMHumFt is correctly assembled as a 24-mer. All the experiments reported in this paper were carried out with ferritin samples derivatized with 12 mM iodoacetic acid.


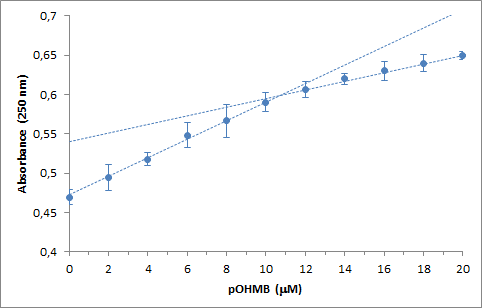


Figure S2: pOHMB titration of S-CMHumFt. The titration was performed on a protein completely reassembled after the treatment with iodoacetic acid (12 mM). Ferritin concentration is 20 μM in monomer. The number of unreacted free thiol groups was determined by titration with hydroxymercuribenzoate (pOHMB) following the mercaptide formation at 250 nm. Once all the thiol groups were titrated, the slight increase in absorbance is due to the contribution of free pOHMB. All the available cysteine residues (about 50%) were titrated at 11 μM pOHMB. Each point represents the mean ± SD (n = 3).

**Stability of S-CMHumFt-Cyt C complex**


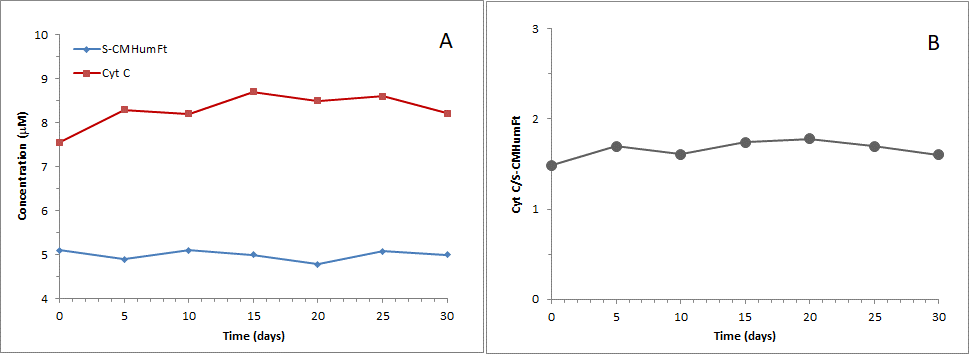


Figure S3: Stability of S-CMHumFt-Cyt C complex monitored by HP-SEC. Panel **A**. Red Line: Cyt C concentration within ferritin cage. Signal was acquired at 410 nm at retention time of ferritin (5.3 min). Blue line: Ferritin concentration acquired at 220 nm at 5.3 min. Panel **B**. Cyt C molecules per ferritin cage. S-CMHumFt-Cyt C complex sterile filtered is stable over a period of 30 days at 4°C. Each point is the mean of two independent measurements.

**Flow cytometry of NB4 cells treated with S-CMHumFt loaded with cytochrome C-AlexaFluor 555**

Figure S4: **A** NB4 cells were treated with S-CMHumFt loaded with cytochrome C-AlexaFluor 555 (Ft-CytC 555), with free cytochrome C-AlexaFluor 555 (cytC 555) or not treated (ctr) for the indicated time points and analyzed by flow cytometry after staining with the vital dye Sytox Blue (SB) which is incorporated only by apoptotic/dead cells. All the cells that incorporated cytochrome C-AlexaFluor 555 (555+) resulted dead (SB+). **B** NB4 cells were treated as in A and with S-CMHumFt alone (Ft) and cell death was analyzed by flow cytometry upon staining with SB. Whereas S-CMHumFt or free cytochrome C-555 had no significant effects, cytochrome C-555 carried by S-CMHumFt induced massive cell death. **C** Flow cytometry dot plots show the uptake of S-CMHumFt-FITC by the entire population of NB4 cells after 24 hours of incubation. **D** NB4 cells were treated (right) or not (left) with S-CMHumFt loaded with cytochrome C-AlexaFluor 555 for 72 hours and analyzed by flow cytometry after staining with the vital dye Sytox Blue (SB). The flow cytometry dot plot shows that all the cells that incorporated cytochrome C-AlexaFluor 555 died, as demonstrated by positivity to SB staining. The figure shows one representative experiment of two.
